# An investigation of aspects of radiochemical purity of ^99m^Tc-labelled human serum albumin nanocolloid

**DOI:** 10.1186/s41181-021-00147-8

**Published:** 2021-10-11

**Authors:** Ruslan Cusnir, Michel Leresche, Claude Pilloud, Marietta Straub

**Affiliations:** Institute of Radiation Physics, Lausanne University Hospital and University of Lausanne, Lausanne, Switzerland

**Keywords:** Nanocolloidal albumin, Lymphoscintigraphy, NanoHSA, Nanotop, ^99m^Tc, Thin-layer chromatography, Radiochemical purity, Glucose, Sep-Pak®

## Abstract

**Background:**

Nanocolloidal human serum albumin radiolabelled with ^99m^Tc provides a diagnostic radiopharmaceutical for sentinel node lymphoscintigraphy. NanoHSA (Nanotop), a commercially available kit, enables the simple preparation of this radiopharmaceutical via reconstitution with pertechnetate eluted from a generator. Thin-layer chromatography is widely used for determining radiochemical purity in clinical nuclear medicine. Quality control methods recommended by the manufacturer were sometimes reported to yield variable results. Therefore, we proposed and evaluated three alternative thin-layer chromatography methods for the quality control of [^99m^Tc]Tc-NanoHSA from a commercially available kit.

**Results:**

The radiochemical purity of [^99m^Tc]Tc-NanoHSA determined with all methods was reproducible and met the requirements of the SPC and the European Pharmacopoeia (≥ 95%). Our quality control using iTLC-SG chromatographic paper in methyl ethyl ketone mobile phase identified only free pertechnetate as impurity, resulting in > 99% RCP. The quality control using iTLC-SG in 85% methanol or iTLC-SA in 0.9% NaCl identified an additional small fraction of a hydrophilic impurity, resulting in 95–97% RCP. Glucose was identified as a potential ^99m^Tc-carrying hydrophilic species contributing to hydrophilic impurities.

**Conclusion:**

Our quality control of [^99m^Tc]Tc-NanoHSA with non-polar mobile phase tended to underestimate the amount of hydrophilic impurities, although without compromising the final quality of the radiopharmaceutical. Alternative TLC methods using aqueous mobile phases enabled a more accurate determination of hydrophilic impurities.

## Introduction

Human serum albumin (HSA) is the most abundant protein in the blood serum and is readily radiolabelled with ^99m^Tc (technetium-99m). Its relatively short half-life (6.02 h) and low-energy gamma emission (141 keV) facilitate diagnostic applications of this radionuclide in a large number of small nuclear medicine centres involving minimal infrastructure. Affordable, commercially available radionuclide generators supply ^99m^Tc as sodium pertechnetate [^99m^Tc]NaTcO_4_, which is compatible with the requirements of Good Manufacturing Practices (GMP) and national pharmacopoeias. The pertechnetate solution eluted from a certified generator is then ready for HSA radiolabelling. The exact nature of how ^99m^Tc binds to HSA is unknown, however the most likely mechanism is the binding of reduced ^99m^Tc to sulfur atoms following the reduction of disulfide bonds of the protein by tin (II) added to the HSA kit (Vanbilloen et al. 1993).

Different physico-chemical forms of HSA labelled with ^99m^Tc enable a range of versatile diagnostic applications (Grosser et al. 2016). Intravenous injection of the soluble [^99m^Tc]Tc-HSA is used as a dynamic blood pool tracer, while slightly denatured, macroaggregated [^99m^Tc]Tc-HSA particles sized 10–100 µm are used for lung perfusion studies. Smaller, 50–80 nm HSA nanoparticles labelled with ^99m^Tc provide a diagnostic radiopharmaceutical agent for sentinel lymph nodes scintigraphy (Giammarile et al. 2013; Sadkin et al. 2020). Several commercially available kit formulations for preparing [^99m^Tc]Tc-HSA nanocolloid have become available recently and are described in the literature (Nanotop®, Nanoalbumon®, Nanocoll®) (Persico et al. 2015; Gommans et al. 2009; Marenco et al. 2021). Since the radiopharmaceutical is prepared shortly prior to administration, a quality control (QC) must be performed immediately after radiolabelling in order to determine the radiochemical purity (RCP). Thin-layer chromatography (TLC) is the most widely used method for QC of radiopharmaceuticals prepared from commercially available kits. Free (unbound) pertechnetate [^99m^Tc]TcO_4_^−^ is the main impurity separated via TLC through a migration with the solvent front, because the nanocolloidal [^99m^Tc]Tc-HSA and colloidal [^99m^Tc]TcO_2_ remain at the origin of the chromatogram. It is essential that at least 95% of the ^99m^Tc is bound to the nanocolloidal HSA before releasing the radiopharmaceutical for administration to patient; this amount must therefore be verified. The 95% RCP benchmark is used as a reference from individual pharmacopoeial monographs of several other ^99m^Tc radiopharmaceuticals. The free pertechnetate [^99m^Tc]TcO_4_^−^ anion is similar to the iodide I^−^ anion and is taken up by sodium/iodide symporter (NIS). This effect can cause an undesired uptake of [^99m^Tc]TcO_4_^−^ into the thyroid, but also into the salivary and gastric tissues, compromising image quality and delivering an unjustified radiation dose to the patient (Hingorani et al. 2010). The efficacy of radiolabelling of the nanocolloidal HSA depends on the size distribution of the particles, however, any excipients such as buffers, inorganic ions, or large amount of glucose present within the kit, as well as the quality of the generator eluate may all play a role in the speciation of ^99m^Tc (Persico et al. 2015; Metaye et al. 2012; Waight et al. 2011).

Several publications covering nanocolloidal human serum albumin include different methods for quality control of the radiolabelled product. The summary of product characteristics (SPC) accompanying the ROTOP-NanoHSA kit recommends two TLC methods for determining the RCP of the final radiopharmaceutical. Method 1 is to use SG-60 aluminium plates as a stationary phase and acetone as a mobile phase. Method 2 is to use iTLC-SA paper as a stationary phase and methyl ethyl ketone as a mobile phase. Both methods enable the migration of free pertechnetate with the solvent front resulting in a retardation factor (R_f_) of 0.9–1, while labelled [^99m^Tc]Tc-NanoHSA remains at the origin, with R_f_ 0.-0.1. These methods are described for RCP determination in different monographs of the European Pharmacopoeia (Council of Europe 2019). The SPC of a similar product called Nanocoll® (GE Healthcare), recently discontinued, recommended 85% aqueous methanol as a mobile phase with iTLC-SA or Whatman No. 1 chromatographic paper, which enables the migration of free pertechnetate with the solvent front yielding R_f_ 0.9–1. As briefly reported elsewhere for [^99m^Tc]Tc-Nanocoll, different TLC methods resulted in varied radiochemical purity for this radiopharmaceutical (Dumas et al. 2009). The SPC of Nanoalbumon® (Medi-Radiopharma Ltd, Erd, Hungary) stipulates 85% aqueous methanol mobile phase with iTLC-SG or Whatman No. 1 for determining the RCP (Marenco et al. 2021). The United States Pharmacopoeia includes a monograph on [^99m^Tc]Tc-albumin colloid injection, giving a method for determining RCP using iTLC-SG with methyl ethyl ketone (United States Pharmacopoeia Convention. United States Pharmacopoeia (USP) 2019).

This study thus aimed at evaluating alternative QC methods for determining the RCP of nanocolloidal ^99m^Tc-labelled HSA prepared from a commercially available kit: NanoHSA (ROTOP Pharmaka AG, Dresden, Germany). In addition to suitability verification, we employed three alternative compendial TLC methods to better separate small hydrophilic impurities. Solid-phase extraction (SPE) using a Sep-Pak® cartridge was used as a complementary method to separate [^99m^Tc]Tc-NanoHSA from hydrophilic impurities, enabling their further analysis with TLC. To evaluate the potential of glucose as a hydrophilic ^99m^Tc-carrying impurity, radiolabelling of glucose with [^99m^Tc]TcO_4_^−^ was tested and analysed with SPE and TLC methods. Finally, we evaluated a range of needles used for QC of radiopharmaceuticals in nuclear medicine departments in order to determine the volume of the spotted sample and its effect on TLC outcome.

## Materials and methods

The drop size produced by syringe needles of different gauges was determined by weighing water drops (n = 5) deposited in a tall glass vial to minimise evaporation (balance XPE205, Mettler Toledo, Columbus, United States). To test the effect of drop size on TLC, 450 MBq of [^99m^Tc]TcO_4_^−^ in 1.5 mL eluate was partially reduced to [^99m^Tc]TcO_2_ in the presence of 40 µg of Sn^2+^. After 10 min incubation at room temperature, samples of 2, 5, 10, 15 and 20 µL in triplicate (n = 3 technical replicates) were spotted onto 1.5 cm × 10 cm iTLC-SG chromatographic paper strips and developed in methyl ethyl ketone. The fraction of free [^99m^Tc]TcO_4_^−^ was determined using a miniGITA TLC scanner (Elysia-Raytest GmbH, Straubenhardt, Germany).The radiolabelling of nanocolloidal human serum albumin was carried out by reconstitution of a commercially available radiopharmaceutical kit: NanoHSA (ROTOP Pharmaka GmbH, Dresden, Germany), according to the instructions provided in the SPC (version 22,509). To verify the suitability of alternative compendial methods for quality control of [^99m^Tc]Tc-NanoHSA, three individual radiolabellings were carried out in identical conditions on three consecutive days. Each NanoHSA kit (batch nr. EP 11 20 11 1 DE, authorisation nr. 905.79.00.00, expiry date 03.2022) was labelled with sodium pertechnetate [^99m^Tc]NaTcO_4_ *Ph. Eur.* at maximum volume (4–5 mL) and maximum activity (4.2–5.5 GBq) eluted from a Pertector-15 generator (Polatom, Otwock, Poland, code MTcG-4, batch 086/20, 15 GBq on 26.10.2020 at 12 h 00). The radiochemical purity of [^99m^Tc]Tc-NanoHSA was determined with thin-layer chromatography using three alternative methods:

*Method A* Stationary phase: 1.5 cm × 10 cm strip of iTLC-SG chromatographic paper, origin at 15 mm, solvent front 70 mm; mobile phase: methyl ethyl ketone.

*Method B* Stationary phase: iTLC-SG chromatographic paper, origin at 15 mm, solvent front 70 mm; mobile phase: methanol/water 85/15 v/v.

*Method C* Stationary phase: iTLC-SA chromatographic paper, origin at 15 mm, solvent front 60 mm; mobile phase: 0.9% aqueous NaCl.

**Fig. 1:**
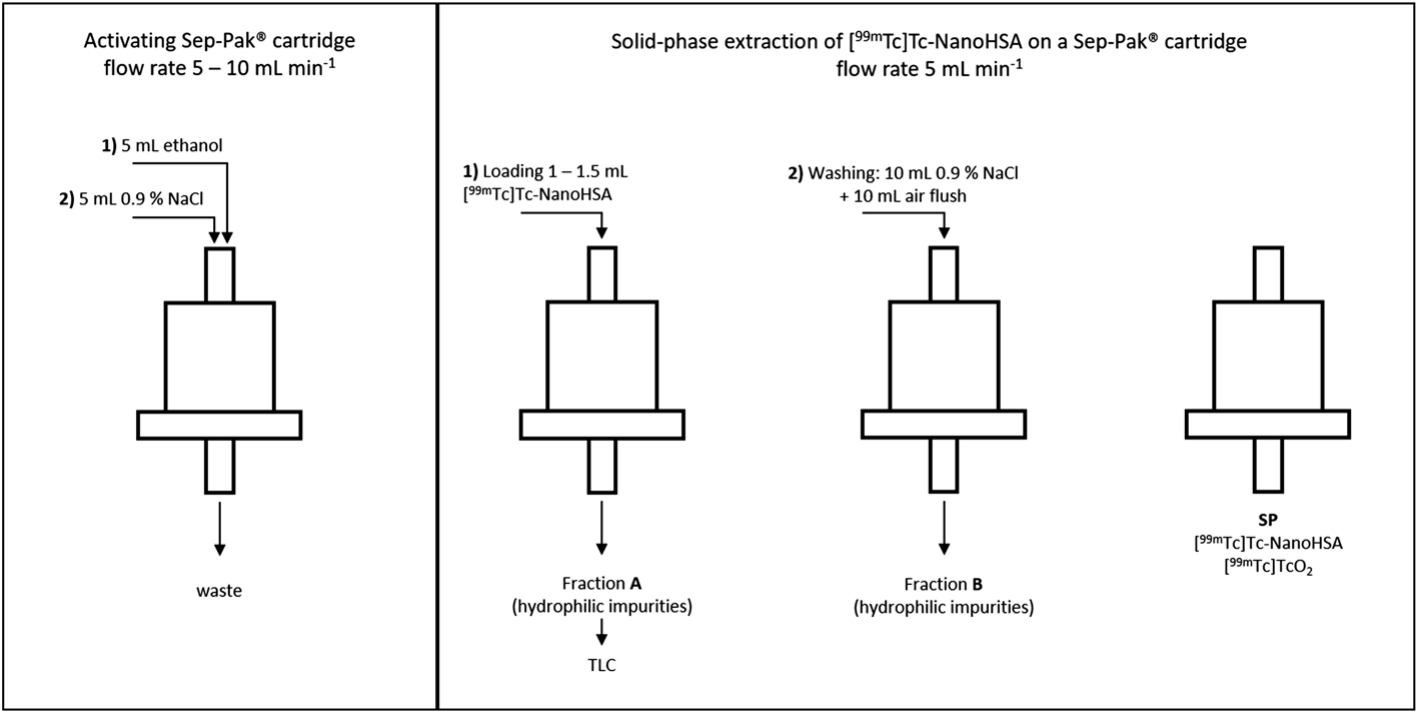
Solid-phase extraction of NanoHSA using Sep-Pak® cartridge. Colloidal [^99m^Tc]TcO_2_ and [^99m^Tc]Tc-NanoHSA were retained on Sep-Pak® cartridge enabling further TLC analysis of soluble hydrophilic impurities in fraction A

For each method, 10 µL of [^99m^Tc]Tc-NanoHSA was deposited on a chromatographic strip immediately after radiolabelling was completed (t_0_, allowing 10 min incubation time according to the SPC), 30 min after radiolabelling (t_1_), and 60 min after radiolabelling (t_2_). The sample drop was not allowed to dry after spotting. Each iTLC was carried out in triplicate (n = 3 technical replicates) for three individual radiolabellings on consecutive days (n = 3 experimental replicates).The radiochemical separation of hydrophilic impurities in [^99m^Tc]Tc-NanoHSA was carried out by solid-phase extraction using Sep-Pak® C18 reversed phase cartridge (Fig. 1). Sep-Pak® cartridge was activated with 5 mL ethanol followed by 5 mL 0.9% aqueous NaCl. Next, 1–1.5 mL [^99m^Tc]Tc-NanoHSA was loaded onto the cartridge and fraction A was collected. The cartridge was washed with 10 mL 0.9% aqueous NaCl followed by 10 mL air flush providing fraction B. The radioactivity of the ^99m^Tc was measured in fraction A, fraction B, and in the cartridge (SP) using a VDC-405 dose calibrator (Veenstra Instruments, Joure, The Netherlands). The radiochemical purity (%) of the [^99m^Tc]Tc-NanoHSA was calculated as follows:

1 $$RCP = \frac{SP \times 100}{{\left( {A + B + SP} \right)}}$$

To identify other hydrophilic impurities in [^99m^Tc]Tc-NanoHSA that were not retained on the Sep-Pak® cartridge, fraction A was analysed additionally using thin-layer chromatography. To determine the free [^99m^Tc]TcO_4_^−^, 10 µL was spotted onto a TLC SG-60 strip on an aluminium support (origin: 15 mm, solvent front: 70 mm) and developed in methyl ethyl ketone. To determine other polar impurities, 10 µL was spotted onto a TLC SG-60 strip (origin: 15 mm, solvent front: 70 mm) and developed in 0.9% aqueous NaCl. To display glucose distribution in the analyte, a sample of fraction A from one NanoHSA radiolabelling was submitted to TLC using a SG-60 strip in methyl ethyl ketone and 0.9% NaCl mobile phases, and after chromatographic separation the strips were developed by briefly dipping in 2% aqueous KMnO_4_. The developed TLC strips were imaged in a UV–visible scanner (Camag, Muttenz, Switzerland).

To evaluate a potential contribution of glucose as a ^99m^Tc-carrying impurity, radiolabelling of glucose was attempted with [^99m^Tc]TcO_4_^−^ in conditions similar to the radiolabelling of the NanoHSA kit. Briefly, 15 mg glucose in 0.9% aqueous NaCl in the presence of 40 µg (in one experiment – 200 µg) of Sn^2+^ was radiolabelled with 0.6–1.5 GBq of [^99m^Tc]TcO_4_^−^. The final volume was adjusted with saline to 1.5–5 mL, and the experiment was reproduced in triplicate (n = 3 experimental replicates). To determine the free pertechnetate, TLC was carried out using SG-60 on an aluminium support as a stationary phase in acetone and in methyl ethyl ketone. To determine other hydrophilic ^99m^Tc species, TLC was carried out with methanol/water 85/15 v/v mobile phase using SG-60 on aluminium and iTLC-SA stationary phase, and with 0.9% NaCl mobile phase using SG-60 on aluminium stationary phase. The distribution of glucose on the TLC strips was revealed by developing the strips in 2% KMnO_4_ and imaging in a UV–visible scanner (Camag, Muttenz, Switzerland). Additionally, after 10–20 min incubation at room temperature, radiolabelled glucose was loaded on a Sep-Pak® cartridge and fraction A was collected. The cartridge was washed with 10 mL 0.9% aqueous NaCl followed by 10 mL air flush providing fraction B. The radioactivity of ^99m^Tc was measured in fraction A, fraction B, and in the cartridge (SP).

## Results

### ***Quality control of ***^***99m***^***Tc-HSA nanocolloid***

#### Effect of needle gauge on TLC performance

To determine the size of a drop deposited for thin-layer chromatography using a syringe, we tested a range of needles of different diameters routinely used for the QC of radiopharmaceuticals in clinical nuclear medicine. As expected, the drop size increased with a larger needle diameter, resulting in drops between 10 µL and 26 µL (Table 1).

**Table 1:** Water drop size as a function of needle diameter. Water density assumed as 1 g mL^−1^

	25G × 5/8″ 0.5 × 16 mm	23G × 1 1/4″ 0.6 × 30 mm	22G × 1 1/4″ 0.7 × 30 mm	21G × 2″ 0.8 × 50 mm	20G × 23/16″ 0.9 × 55 mm	19G × 1 1/2″ 1.1 × 40 mm	18G × 1 1/2″ 1.2 × 40 mm
m, g *(n* = *5)*	0.0100	0.0146	0.0156	0.0170	0.0171	0.0202	0.0264
V, µL	10	14.6	15.6	17	17.1	20.2	26.4
SD, ± g *(k* = *2)*	0.0020	0.0043	0.0040	0.0023	0.0012	0.0021	0.0023

We then determined the fraction of free [^99m^Tc]TcO_4_^−^ as a function of volume spotted following a partial reduction with Sn^2+^. A range of samples from 2 µL to 20 µL spotted on iTLC-SG paper and developed in methyl ethyl ketone showed that radiochemical yield of free [^99m^Tc]TcO_4_^−^ appeared to decrease with a larger drop size (Table 2).

**Table 2:** Fraction of free [^99m^Tc]TcO_4_^−^ determined with iTLC as a function of drop size

Drop volume	2 µL	5 µL	10 µL	15 µL	20 µL
% free [^99m^Tc]TcO_4_^−^	1.57 ± 0.20	0.60 ± 0.11	0.62 ± 0.15	0.29 ± 0.04	0.23 ± 0.03

Each experiment was carried out in triplicate (n = 3) using iTLC-SG strips developed in MEK mobile phase

#### Thin-layer chromatography

We tested three alternative compendial methods to differentiate radiochemical species in [^99m^Tc]Tc-NanoHSA. iTLC-SG paper as stationary phase was used with MEK and 85% methanol mobile phases, and iTLC-SA paper with 0.9% NaCl. Our quality control results obtained with iTLC showed that the radiochemical purity of [^99m^Tc]Tc-NanoHSA in all radiolabellings fulfilled the requirements set in the SPC (≥ 95%, Table 3). iTLC with methyl ethyl ketone as mobile phase showed consistent and reproducible quantitative radiolabelling at all time points from t_0_ to 60 min. Conversely, iTLC with aqueous mobile phases indicated lower RCP compared to MEK after 10 min incubation: 96.2 ± 0.3% in 85% methanol and 96.5 ± 0.3% in 0.9% NaCl, against 99.7 ± 0.2% in MEK. The RCP determined with 85% methanol and with saline mobile phases was similar and showed a slight increase between t_0_ (96.2 ± 0.3% and 96.5 ± 0.3% respectively) and 30 min incubation time (98.1 ± 0.4% and 98.0 ± 0.1% respectively).

**Table 3:** Radiochemical purity of [^99m^Tc]Tc-NanoHSA. RCP determined with iTLC and SPE for three individual radiolabellings (n = 3) carried out on three consecutive days

	MEK iTLC-SG	85% Methanol / H_2_O iTLC-SG	0.9% NaCl iTLC-SA	Sep-Pak®
t_0_	99.7 ± 0.2	96.2 ± 0.3	96.5 ± 0.3	95.7 ± 0.1#
+ 30 min	99.9 ± 0.1	98.1 ± 0.4	98 ± 0.1	97.6 ± 0.0*
+ 60 min	100 ± 0.1	98.6 ± 0.1	98.7 ± 0.0	98.3 ± 0.1*

^#^n = 3 Sep-Pak® experimental replicates *n = 2 Sep-Pak® experimental replicates

#### Solid-phase extraction

We used solid-phase extraction on a Sep-Pak® C18 reversed phase cartridge to further separate and identify potential hydrophilic impurities from the [^99m^Tc]Tc-NanoHSA. The RCP of the [^99m^Tc]Tc-NanoHSA determined with a Sep-Pak® cartridge (Table 3) was in good agreement with the iTLC results obtained in aqueous mobile phases (85% methanol and saline), but not in MEK. We observed a similar trend towards an increase of RCP with an incubation time of over 30 min. Additional TLC analysis of the fraction A collected from the Sep-Pak® cartridge enabled a separation of hydrophilic impurities into several species. The TLC results for fraction A (Table 4) showed that the distribution of different ^99m^Tc species varied in different experiments with a 20–50% standard deviation. Such variability is typical for non-specific impurities, which are rarely reproducible with a high degree of accuracy. According to TLC results obtained in MEK mobile phase (Table 4), about 50% of the fraction A contained free pertechnetate [^99m^Tc]TcO_4_^−^, with the remaining 50% attributed to colloidal [^99m^Tc]TcO_2_ and/or other polar hydrophilic impurities that did not migrate in MEK. However, TLC of the same sample in 0.9% NaCl mobile phase identified only 24–25% of colloidal [^99m^Tc]TcO_2_ (Table 4). Collating these results suggests that approximately 25% of the fraction A, or 1–2% of the total radioactivity in [^99m^Tc]Tc-NanoHSA, represent a different polar species carrying ^99m^Tc, other than free pertechnetate.

**Table 4:** Radiochemical speciation of hydrophilic impurities in [^99m^Tc]Tc-NanoHSA

	Sep-Pak® fraction A		
	MEK	0.9% NaCl	
	% [^99m^Tc]TcO_4_^−^	% [^99m^Tc]TcO_2_	% polar impurities
t_0_*	49.4 ± 8.2	24.1 ± 8.4	75.9 ± 8.4
+ 30 min#	53.4 ± 22.7	20.1 ± 7.2	79.9 ± 7.2
+ 60 min#	48.0 ± 16.9	25.7 ± 12.4	74.3 ± 12.4

Determined in fraction A, containing hydrophilic impurities not retained on the Sep-Pak® cartridge, with TLC SG-60 stationary phase

^*^n = 3 experimental replicates

^#^n = 2 experimental replicates

#### *Radiolabelling glucose with [*^*99m*^*Tc]TcO*_*4*_^*−*^

Thin-layer chromatography of glucose in the presence of [^99m^Tc]TcO_4_^−^ identified hydrophilic species carrying ^99m^Tc capable of migrating with the solvent front in aqueous mobile phases (Table 5). Free pertechnetate fraction separated with acetone (16.0%) and MEK (25.7%) mobile phases was higher in the experiment with a high activity concentration of ^99m^Tc (1.5 GBq in 2 mL), compared to 3.3% in acetone and 3.1% in MEK when glucose was radiolabelled with less ^99m^Tc (0.06 GBq in 1.5 mL). Furthermore, the free [^99m^Tc]TcO_4_^−^ fraction appeared to decrease with incubation time from t_0_ to 1 h. The TLC in aqueous mobile phases identified a consistently higher fraction of mobile ^99m^Tc-carrying species in both low activity concentration (36.1–39.0%) and high activity concentration (36.1–53.8%) radiolabellings. Again, the fraction of mobile ^99m^Tc-carrying species decreased from 36.1–39.0% to 12.4–12.9% within 1 h incubation time. The radiolabelling of glucose attempted in the presence of 200 µg of Sn^2+^ resulted entirely in colloidal [^99m^Tc]TcO_2_ immobilised at the origin on TLC strips.

**Table 5:** Fraction (%) of mobile ^99m^Tc species in radiolabelled glucose determined with TLC

	[1]	[2]	[1]	[2]	[1]	[2]	[1]	[2]	[1]	[2]
GBq mL^−1 99m^Tc	0.75	0.04	0.75	0.04	0.75	0.04	0.75	0.04	0.75	0.04
TLC conditions	TLC SG-60 Acetone 30–35 min		TLC SG-60 MEK 14–15 min		TLC-SA 85% methanol 14–15 min		TLC SG-60 85% methanol 30–32 min		TLC SG-60 0.9% NaCl 14–15 min	
Identification	Free [^99m^Tc]TcO_4_^−^		Free [^99m^Tc]TcO_4_^−^		Free [^99m^Tc]TcO_4_^−^ and ^*99m*^*Tc-glucose*		Free [^99m^Tc]TcO_4_^−^ and ^*99m*^*Tc-glucose*		Free [^99m^Tc]TcO_4_^−^ and ^*99m*^*Tc-glucose*	
t_0_	16.0	3.3	25.7	3.1	53.8	39.0	36.1	36.1	48.1	36.5
+ 30 min		1.6		2.2		19.3		17.2		19.1
+ 60 min		2.2		2.0		12.9		12.4		12.5

Determined for two individual radiolabellings with different activity concentration of ^99m^Tc (15 mg glucose, 40 µg Sn^2+^, 1.5–2 mL of 0.9% NaCl)

**Fig. 2:**
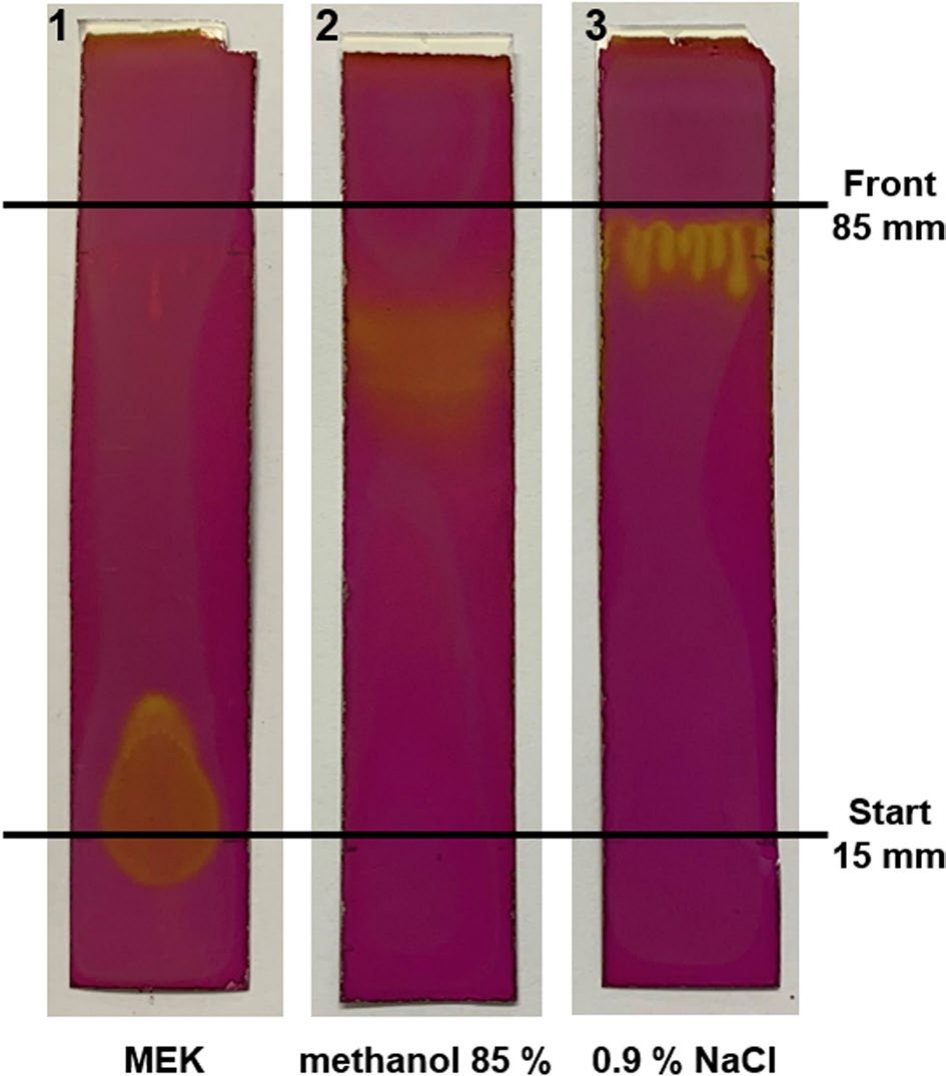
Distribution of glucose on TLC SG-60 strips. Glucose (yellow spot) migrated with the solvent front in 0.9% NaCl and in 85% methanol, and remained at the origin in MEK

The distribution of glucose using different mobile phases on TLC plates developed in 2% KMnO_4_ showed that glucose remained at the origin in MEK (Fig. 2, strip 1), and in acetone (not shown). When TLC was carried out in 85% methanol or in saline, the glucose migrated with the solvent front (Fig. 2, strip 2 and 3).

The solid-phase extraction of glucose radiolabelled with [^99m^Tc]TcO_4_^−^ showed that glucose was not retained on the C18 Sep-Pak® cartridge. A nearly equal distribution of ^99m^Tc radioactivity between fraction A collected upon loading and fraction B collected upon washing the cartridge speaks in favour of a weak non-specific retention of soluble ^99m^Tc species (Table 6). Approximately 30–40% of the ^99m^Tc was not released from the cartridge upon washing and was attributed to colloidal [^99m^Tc]TcO_2_.

**Table 6:** Distribution of [^99m^Tc]Tc-glucose on Sep-Pak® cartridge

	[1] % ^99m^Tc	[2] % ^99m^Tc	[3] % ^99m^Tc	Identification
Fraction A	47.2	30.6	34.9	Free [^99m^Tc]TcO_4_^−^ and *[*^*99m*^*Tc]Tc-glucose*
Fraction B	21.7	29.1	28.5	
A + B	68.9	59.7	63.4	
Residual ^99m^Tc Sep-Pak®	31.1	40.3	36.6	Colloidal [^99m^Tc]TcO_2_

Determined for three individual experiments (15 mg glucose, 40 µg Sn^2+^, 4 mL of 0.9% NaCl)

## Discussion

### Effect of needle gauge on TLC performance

The amount of a sample deposited on the chromatographic support for thin-layer chromatography is essential for the efficient separation of species present in a radiopharmaceutical. Different SPCs and pharmacopoeial monographs recommend spotting between 2 and 5 µL, and up to 10 µL per sample. Small nuclear medicine centres which employ radiopharmaceutical kits often do not have a laboratory equipped with pipettes that are checked and calibrated on a regular basis. Thus, quality control of radiopharmaceuticals is performed by withdrawing a small volume of radiolabelled product using a sterile syringe and needle and spotting a drop on an iTLC strip. The drop size distribution as a function of needle gauge summarised in Table 1 showed that the smallest 25G needle produced a 10 µL drop, which is the largest amount of sample recommended for iTLC by QC guidelines. In clinical nuclear medicine, needle sizes ranging from gauge 20G to 26G are used, and are routinely available to professionals carrying out the QC of [^99m^Tc]Tc-NanoHSA. Needles 25G or 27G are recommended for subcutaneous injections for lymphoscintigraphy with radiotracers which include nanocolloidal HAS (Bluemel et al. 2015). In this study, only a 25G needle was shown to provide 10 µL, equivalent to the volume deposited with a pipette. This means that caution should be taken when interpreting iTLC results obtained with larger needles.The determination of free pertechnetate in partially reduced eluate as a function of volume spotted for iTLC showed a trend towards an apparent decline of the free pertechnetate fraction with increased sample volume: from 1.57 ± 0.20% when spotted with 2 µL and up to 0.23 ± 0.03% when spotted with 20 µL. These small numbers obtained with iTLC do not provide the most robust conclusions regarding the effect of sample volume on chromatographic separation and should be considered indicative results only. However, it must be noted that chromatograms of larger sample volumes – with 15 and 20 µL spots – had to be analysed with the TLC scanner once they had been left to decay by one half-life. When using the same settings as for the 2–10 µL spots immediately after developing the strips, the 15 and 20 µL spots saturated the radioactivity detector, thus compromising the quantification.

### Thin-layer chromatography

According to the SPC, the methods recommended for determining the RCP of [^99m^Tc]Tc-NanoHSA employ thin-layer chromatography with SG-60 on aluminium stationary phase in acetone as mobile phase, or iTLC-SA stationary phase in methyl ethyl ketone. For both methods ^99m^Tc-labelled human serum albumin nanocolloid remains at the origin, while free unbound pertechnetate [^99m^Tc]TcO_4_^−^ migrates with the solvent front. These methods are widely recommended in the literature. However, for some time now, acetone has not been recommended for this purpose because it tends to overestimate the free [^99m^Tc]TcO_4_^−^ fraction due to its higher water content (Decristoforo and Zolle 2007). Methyl ethyl ketone is the ultimate mobile phase to enable an accurate separation of free pertechnetate with TLC (World Health Organisation 2019).

Therefore, we selected three alternative methods and verified their suitability for the QC of [^99m^Tc]Tc-NanoHSA in a coherent, reproducible fashion with a minimal number of replicates. We chose iTLC-SG chromatographic paper for MEK mobile phase because this stationary phase is more widely used in practise compared to iTLC-SA, and it is easier to handle because it does not require thermal activation prior to use, unlike iTLC-SA. Additionally, we chose methanol/water 85/15 mobile phase for iTLC-SG because it enables the migration of small hydrophilic species along with the free pertechnetate [^99m^Tc]TcO_4_^−^. Methanol may contribute to the chemical degradation of delicate biological molecules such as human serum albumin. Therefore, iTLC was also carried out in a 0.9% NaCl solution as mobile phase with iTLC-SA stationary phase (World Health Organisation. The International Pharmacopoeia. Technetium (^99m^Tc) pentetate complex injection (Technetii (^99m^Tc) pentetatis multiplex injectio); 2019.

The QC of the ^99m^Tc-labelled NanoHSA using MEK showed quantitative RCP in all radiolabellings, with the free [^99m^Tc]TcO_4_^−^ fraction below 1% shortly after reconstitution of the kit. To determine the presence of other potential soluble impurities, more polar, aqueous mobile phases are necessary. iTLC-SG in 85% methanol showed quantitative radiolabelling of NanoHSA, however a slightly lower RCP after 10 min incubation (96.2 ± 0.3%). After 30 min incubation the RCP increased to over 98%. Although the initial result fulfilled the QC requirements of the SPC, it suggested the presence of a labile hydrophilic species carrying ^99m^Tc that did not migrate in MEK. iTLC-SA in 0.9% NaCl showed remarkably similar results with methanol/water (Table 3). The RCP after 10 min incubation was 96.5 ± 0.3%, comparable to methanol/water (96.2 ± 0.3%) but slightly lower than with MEK (99.7 ± 0.2%). After 30 min incubation, the RCP increased to 98%, same as methanol/water. A comparison of the results of three iTLC methods revealed that MEK overestimated the RCP of [^99m^Tc]Tc-NanoHSA, identifying only free pertechnetate impurity. Aqueous mobile phases identified approximately 1–2% of additional hydrophilic impurities, which consistently tended to decline within the timeframe of experiment (10–60 min).

### Solid-phase extraction

Solid-phase extraction is used as an alternative to TLC for the quality control of some radiopharmaceuticals. Sep-Pak® cartridges with C18 hydrophobic reversed phase retain non-polar compounds and particles (Straub et al. 2018; Ramirez et al. 2000). Colloidal [^99m^Tc]TcO_2_ is also retained on the Sep-Pak® and we hypothesised that larger colloidal particles (≤ 80 nm) of human serum albumin labelled with ^99m^Tc could also be retained on the cartridge. The RCP of [^99m^Tc]Tc-NanoHSA determined with SPE was similar to iTLC in methanol/water and in saline (Table 3), showing that Sep-Pak® makes it possible to separate the same hydrophilic impurities. Furthermore, this method may be used as a less expensive and rapid alternative for QC in the absence of a TLC scanner. However, the manual handling of SPE cartridges during this QC can result in a higher radiation dose to the operator and should be avoided. Furthermore, uncontrolled, manual elution may result in inaccurate and irreproducible results due to variations in the speed of elution.

### ***Identification of a hydrophilic ***^***99m***^***Tc impurity***

Examining the results of the TLC and SPE of [^99m^Tc]Tc-NanoHSA suggested the presence of a labile hydrophilic species carrying ^99m^Tc. Although such species in no way impairs the final quality of the radiopharmaceutical, it is of interest to speculate on its nature. NanoHSA from ROTOP Pharmaka is a freeze-dried radiopharmaceutical kit containing 0.5 mg of colloidal particles of human serum albumin. According to the SPC, after reconstituting the kit, 95% of the particles sized ≤ 80 nm are radiolabelled with ^99m^Tc. The excipients present in the kit include: tin chloride dihydrate as a reducing agent, glucose as a filling agent, an antifoaming agent called poloxamer 238, a sodium phosphate di-hydrate buffering agent, and a sodium phytate chelating agent for scavenging unbound ^99m^Tc.Considering the chemical properties of each excipient, the hypothetical ^99m^Tc-labelled species can be narrowed down to phytate or glucose. Phytic acid is a strong chelating molecule that forms stable complexes with multivalent transition metal ions (Vasca et al. 2002). Applications of phytate labelled with ^99m^Tc have been reported in the literature, and a radiopharmaceutical kit (Phytacis®, IBA Molecular) is commercially available for diagnostic applications (Rao et al. 1990; Tavares et al. 2001; Takei et al. 2006). The chromatographic behaviour of [^99m^Tc]Tc-phytate does not exhibit migration with solvent front in methanol/water mobile phase according to the QC method described in the SPC. Thus, [^99m^Tc]Tc-phytate can be excluded as a hydrophilic impurity observed in this study.

**Fig. 3:**
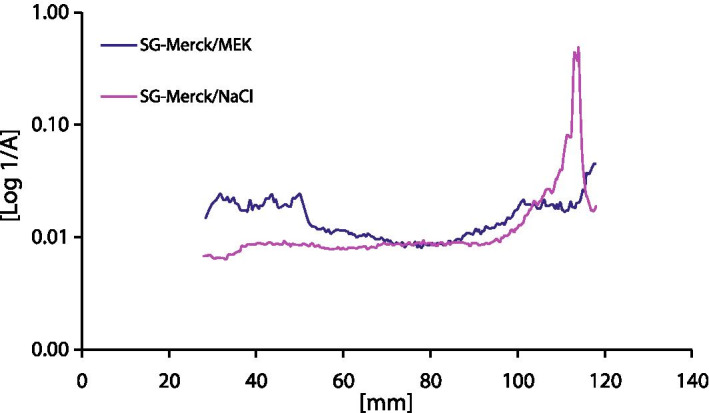
Distribution of glucose on a TLC of the hydrophilic fraction of [^99m^Tc]Tc-NanoHSA. [^99m^Tc]Tc-NanoHSA from one radiolabelling was retained on a Sep-Pak® cartridge and hydrophilic fraction A was submitted to TLC. Glucose migrated with the solvent front in 0.9% NaCl (purple trace) and mostly remained at the start in MEK (blue trace) mobile phase

Native non-functionalised glucose is only a very weak ligand for transition metals, and there are no stable complexes reported with ^99m^Tc (Klufers and Kunte 2001; Bowen and Orvig 2008). Furthermore, ^99m^Tc chemistry exhibits a strong preference towards N and S atoms when forming radiolabelled compounds (Alberto et al. 2020). Nevertheless, the imaging of glucose distribution on a TLC of the hydrophilic fraction A separated from [^99m^Tc]Tc-NanoHSA using a Sep-Pak® cartridge showed that the glucose followed the distribution of ^99m^Tc radioactivity in 0.9% NaCl and in 85% methanol mobile phases (Figs. 2 and 3). A large excess of glucose in the kit (15 mg) compared to nanocolloidal albumin (0.5 g) and ^99m^Tc (1 GBq of ^99m^Tc equivalent to 5 × 10^–9^ g) could enable the formation of a transient, elusive complex of [^99m^Tc]Tc-glucose, sufficient to be picked up with TLC. This type of intermediate reaction supports our finding that the RCP of [^99m^Tc]Tc-NanoHSA tended to increase between 10 and 60 min incubation time. In experiments with radiolabelling of glucose, the fraction of mobile ^99m^Tc-carrying species decreased from 39 to 12% within 1 h incubation time (Table 5), which can be attributed to the formation of insoluble [^99m^Tc]TcO_2_ colloid owing to dissociation of the labile [^99m^Tc]Tc-glucose compound. Furthermore, varying distribution of radioactivity in the different fractions following the SPE of radiolabelled glucose (Table 6) showed an irreproducible behaviour, which was expected as a result of the weak, non-specific interaction of glucose with ^99m^Tc. Another alternative explanation may be that a [^99m^Tc]Tc-gluconate complex forms when a small fraction of the glucose in the kit becomes oxidised to gluconic acid.

## Conclusions

The quality control of a commercially available NanoHSA kit labelled with ^99m^Tc showed a reproducible RCP that fulfilled the criteria of the SPC, and the minimal 95% RCP requirement recommended by the European Pharmacopoeia for ^99m^Tc-radiopharmaceuticals. A slight divergence was observed between polar and non-polar mobile phases used with iTLC. Thin-layer chromatography with polar aqueous mobile phases identified the presence of a small fraction of hydrophilic ^99m^Tc-carrying species, potentially a weak labile complex of glucose, which contributes to overestimating the RCP in non-polar mobile phases. Solid-phase extraction using a Sep-Pak® cartridge yielded a RCP comparable to TLC with methanol/water and saline mobile phases and made it possible to separate hydrophilic impurities from the nanocolloidal [^99m^Tc]Tc-HSA. A further TLC analysis of this SPE-separated hydrophilic fraction identified the presence of a hydrophilic impurity other than free pertechnetate. The chromatographic behaviour of glucose in the presence of [^99m^Tc]TcO_4_^−^ supported our hypothesis of a ^99m^Tc-carrying glucose species as a potential hydrophilic impurity in the [^99m^Tc]Tc-NanoHSA. Finally, nuclear medicine professionals running iTLC in a clinical setting should be aware of a potential misinterpretation of the RCP when using larger needles for spotting the samples.

## Data Availability

Data can be obtained upon request.

## Abbreviations

GMP: Good manufacturing practices
HSA: Human serum albumin
iTLC: Instant thin-layer chromatography
MEK: Methyl ethyl ketone
NIS: Sodium/iodide symporter
QC: Quality control
R_f_: Retardation factor
RCP: Radiochemical purity
SPC: Summary of product characteristics
SPE: Solid-phase extraction
TLC: Thin-layer chromatography
